# Activated Ductile CFRP NSMR Strengthening

**DOI:** 10.3390/ma14112821

**Published:** 2021-05-25

**Authors:** Jacob Wittrup Schmidt, Christian Overgaard Christensen, Per Goltermann, José Sena-Cruz

**Affiliations:** 1 Department of Built Environment, University of Aalborg, 9220 Aalborg, Denmark; 2 Department of Civil Engineering, Technical University of Denmark, 2800 Kongens Lyngby, Denmark; coch@byg.dtu.dk (C.O.C.); pg@byg.dtu.dk (P.G.); 3 Department of Civil Engineering, ISISE/IB-S, University of Minho, 4800-058 Guimarães, Portugal; jsena@civil.uminho.pt

**Keywords:** CFRP, strengthening, ductility, post-tensioning, concrete structures

## Abstract

Significant strengthening of concrete structures can be obtained when using adhesively-bonded carbon fiber-reinforced polymer (CFRP) systems. Challenges related to such strengthening methods are; however, the brittle concrete delamination failure, reduced warning, and the consequent inefficient use of the CFRP. A novel ductile near-surface mounted reinforcement (NSMR) CFRP strengthening system with a high CFRP utilization is introduced in this paper. It is hypothesized that the tailored ductile enclosure wedge (EW) end anchors, in combination with low E-modulus and high elongation adhesive, can provide significant strengthening and ductility control. Five concrete T-beams were strengthened using the novel system with a CFRP rod activation stress of approximately 980 MPa. The beam responses were compared to identical epoxy-bonded NSMR strengthened and un-strengthened beams. The linear elastic response was identical to the epoxy-bonded NSMR strengthened beam. In addition, the average deflection and yielding regimes were improved by 220% and 300% (average values), respectively, with an ultimate capacity comparable to the epoxy-bonded NSMR strengthened beam. Reproducible and predictable strengthening effect seems obtainable, where a good correlation between the results and applied theory was reached. The brittle failure modes were prevented, where concrete compression failure and frontal overload anchor failure were experienced when failure was initiated.

## 1. Introduction

Pultruded carbon fiber-reinforced polymers (CFRPs) for near-surface mounted reinforcement (NSMR), as a means to strengthen concrete structures, has been used for several decades (e.g., [1,2,3,4,5,6,7,8,9,10]). The strengthening often results in a significant amount of benefits, namely lower additional permanent loads (CFRP is ~80% lighter than steel), possibility of mobilizing higher tensile strength (CFRP systems are approximately five times stronger than steel), excellent durability properties, installation easiness, among others.

However, several additional failure modes, from those already known from un-strengthened concrete structures, may arise when using CFRP strengthening, such as IC (intermediate crack) de-bonding [11,12,13,14,15,16] and concrete cover separation [17,18]. Consequently, CFRP strengthening is often at the expense of a significantly reduced ductility compared to the un-strengthened concrete structure. These applications are; thus, often challenged by a more general discussion related to lack of failure warning and expected safety margin.

A way to further utilize the CFRP as well as increase the ductility of the strengthened elements may be based on the use of more ductile anchorage zones along the CFRP laminate [19,20,21,22]. However, these systems are more related to CFRP plate and sheet systems. Similar for these systems is; however, still the low magnitude of ductility provided by the systems compared to the un-strengthened reference beam. In addition, limited solutions are commercially available in the field of CFRP plate and sheet strengthening and seems even less available for CFRP rod strengthening systems [23,24].

CFRP rods can experience different types of failure modes when mechanically anchored for NSMR CFRP strengthening systems [25,26,27]. These failures are due to the anisotropic nature of the CFRP material, which provides weaker properties in the transverse direction of the fibers as well as brittleness. Premature mechanical anchorage failure modes, due to crushing of the CFRP, soft slip, power slip, cutting of the fibers, bending of fibers, frontal overload, and fiber failure [27], are thus often experienced. Fiber failure and power slip are typically reached of higher load magnitudes. Control of the stress development along the anchorage length seems to be the key aspect to increase utilization of the CFRP [21].

Mechanical anchoring and activation of a CFRP NSMR system in a bonded configuration have shown to provide high material utilization (e.g., [28,29]). Activation of 50% (approximately 980 MPa) and 70% (approximately 1415 MPa) of the tensile capacity of the CFRP rod was applied before adhesively bonding the system to the concrete structure. Consistent results as well as high levels of strengthening efficiency were obtained, where CFRP rupture was experienced at approximately 3300 MPa (for the 70% activated configuration).

This stress magnitude corresponds to an approximate ultimate strain magnitude of 0.02 mm/mm. Consequently, the method provided better utilization of the CFRP compared to un-activated freely-bonded CFRP strengthening systems and seemed beneficial. It is; however, still an open question whether this CFRP strain magnitude combined with brittle failure modes still provides a sufficient safety margin and failure warning. Increased ductility seems possible if introducing a flexible system which allows for increased elongation in the bonded zone of the CFRP rod and at the anchorage zones. In an unbonded configuration, the CFRP rod is free to elongate along its entire length, thus resulting in increased ductility [30].

However, in such a system the CFRP material is exposed, losing some of the advantages of the NSMR; moreover, the anchorage zones are subjected to the full load regime during the structural service life.

This paper presents a strengthening system where a novel ductile anchorage [30] is used to anchor 8 mm CFRP rods in an NSMR strengthening setup. Flexible adhesive is used, replacing typical stiff epoxy adhesives, thus facilitating a larger elongation provided by the system. It is hypothesized that the system provides a combination of a high strengthening magnitude and significantly increased ductility to the concrete element (in this case T-section reinforced concrete (RC) beams). The system is additionally deemed to enable a wider stress distribution in the adhesively-bonded interface, thus reducing or even preventing a brittle behavior. Finally, a tailorable response control provided by the system components is assumed to enable a high control of stresses and thus a good result consistency.

Consequently, it is hypothesized that the ductile system response provides significantly increased ductility compared to conventional epoxy-bonded NSMR strengthening systems. An additional scope is to provide a strengthening system that provides high CFRP utilization but with a more controlled response and non-brittle failure mode compared to other CFRP strengthening systems. According to the authors’ best knowledge, the researched system has not been proposed before and constitutes; therefore, a further major step towards providing ductility and non-brittle failure modes to RC elements strengthened with CFRP materials.

An experimental program which includes five concrete T-beams strengthened with the novel system is; thus, presented in the paper, where a CFRP rod activation stress of approximately 980 MPa is applied. The unique ductile CFRP system behavior and mounting procedure, as well as the ductile anchor behavior during beam loading, is evaluated experimentally and compared to analytical theoretical predictions.

## 2. Experimental Program

### 2.1. RC T-Section Beams and Test Setup

**Figure 1:**
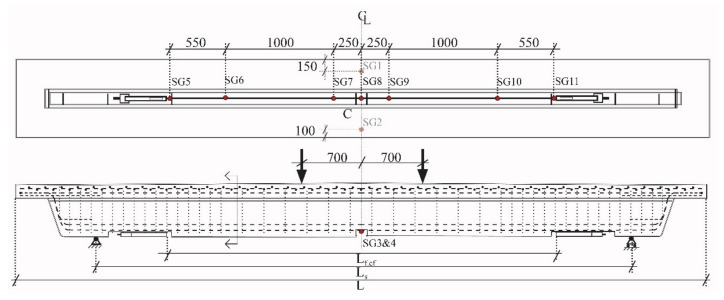
Concrete T-beam dimensions, load setup, and applied monitoring. Note: Units in mm.

**Figure 2:**
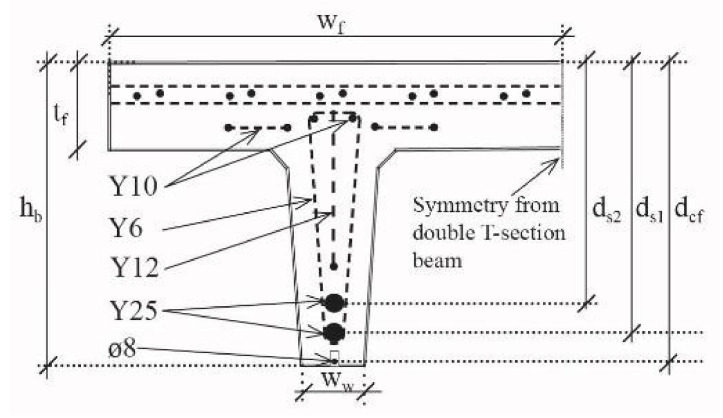
Cross section measures and reinforcement location.

Figure 1 and Figure 2 shows details about the T-section RC beams used for investigating the activated ductile CFRP NSMR strengthening. The beam length was 6.4 m with a support distance of 5 m and a distance of 1.4 m between the two loading points in the four-point-bending configuration. Six beams were quasi-statically loaded until failure with a deformation-controlled load rate of 2 mm/min. Concrete strain gauges, Strain gauge 1 (SG1) and Strain gauge 2 (SG2), (type: 1-LY41-50/120) were placed on the top flange surface at the transverse center line. Two steel strain gauges, Strain gauge 3 (SG3) and Strain gauge 4 (SG4), (type: 1-LY416/120) were placed on the exposed bottom reinforcement steel in the groove. Seven strain gauges (Strain gauge 5 (SG5) to Strain gauge 11 (SG11)) were placed on the CFRP rod using three different types (Types: 1-LD20-6/120, 1-LD20-6/350, and 1-LD2010/350). Linear Variable differential transformers (LVDTs) (Novotechnik position transducer; repeatability ±0.002 mm, Novotechnik, Southborough, MA, USA) were used to measure deformations at the quarter points, at the supports, and at the center location. A data acquisition frequency of 1 Hz was used. Figure 1 depicts the cross section geometry, the reinforcement location, and where the anchor blocks with the ductile anchorage were installed, approximately 1800 mm from the center line.

The longitudinal tensile reinforcement consisted of 2Ø25 deformed steel reinforcement bars, and the top reinforcement consisted of longitudinal Ø10 bars as well as two reinforcement grids. The Ø6 stirrups were spaced by 100 mm and with a double spacing after 1.4 m from the extremities until the beam middle (see Figure 1). More detailed information concerning the beam can be found in [29]. However, relevant geometrical parameters of the T-beams are presented in Table 1.

**Figure 3:**
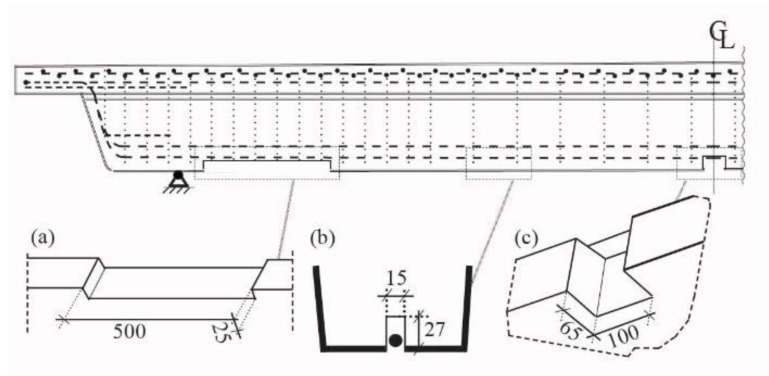
Groove dimensions used for (**a**) anchorage zone, (**b**) Near surface mounted reinforcement (NSMR), and (**c**) mid-span notch. Note: units in mm.

Three different grooves were cut in the concrete beam before applying the ductile CFRP NSMR strengthening system: (i) Anchor block groove (Figure 3a), (ii) groove in which the NSMR rod was mounted (Figure 3b), and (iii) notch located at the mid-span (Figure 3c).

The mid-span notch had a width of 100 mm and a depth of 65 mm, which ensured exposure of the internal Y25 steel reinforcement and thus enabled mounting of strain gauges. In addition, a groove width of 15 m with a depth of 27 mm was cut into the web bottom concrete cover, along the beam length, in order to allow NSMR mounting. The depth was ensured relatively large to provide a good support for the CFRP rod mounted into the more flexible low E-modulus adhesive, and allow for a more desirable distribution of stresses. Finally, the anchor block groove ensured a 4 mm cover layer on the CFRP rod, using a width and depth of 500 and 25 mm, respectively.

### 2.2. Activation of the System and Mounting Procedure

**Figure 4:**
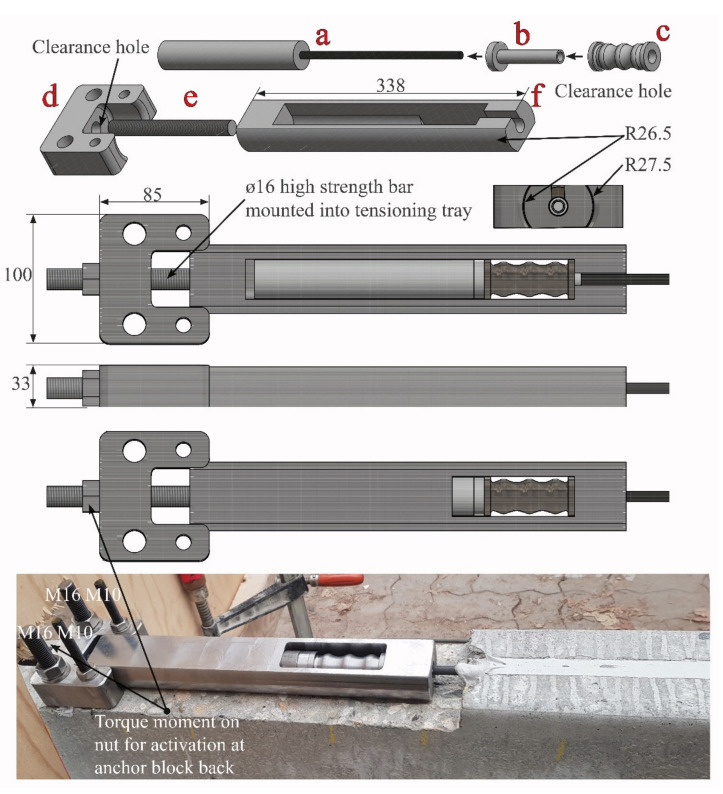
Anchor block used for the ductile Enclosure Wedge (EW) anchor. Note: Units in mm.

Figure 4 depicts the installed anchor block at the ends of a CFRP NSMR strengthening system. The assembled anchor system consists of the following components: (a) Enclosure wedge (EW) anchor, (b) response control pin (RCP), (c) ductile mechanism, (d) anchor block, (e) high strength M16 threaded activation bar, and (f) tensioning tray [29]. The clearance hole in the anchor block allows the movement of the tensioning tray thus enabling activation of the anchored NSMR system. The existing clearance in the tensioning tray provides the space for the response control pin to deform with the ductile mechanism and thus control the deformation.

In the activated ductile system, the 100 × 85 × 33 mm^3^ anchor block was mounted on the concrete beam using two M16 and M10 adhesively-anchored M8.8 threaded bars. The anchor block provides the base for the high strength M16 activation bar mounted into the 338 × 53 mm^2^ tensioning tray. The threaded bar, on which the activation nut was mounted, penetrates through an Ø17 clearance hole in the anchor block and additionally stabilizes the tensioning procedure. In addition, this stabilization is improved by using circular side faces of the tensioning tray, which fits into the indent in the anchor block. By tightening the activation nut, the activation of the ductile-anchored NSMR CFRP strengthening system is enabled.

The strengthening system mounting procedure involves: (i) Cutting of grooves for anchor block and CFRP NSMR slit into the concrete beam, (ii) installation of the anchor block on the RC beam, (iii) mounting the ductile EW anchor on the CFRP rod, (iv) placing of the mounted anchor in the tensioning tray, (v) injection of adhesive in the slit bottom, (vi) inserting the tension tray into the anchor block, (vii) activation of the CFRP rod through a torque moment on the nut applied to the M16 threaded high strength bar, and (viii) final injection of the adhesive to fully cover the CFRP rod in the NSMR system.

### 2.3. Ductile Anchor System

**Figure 5:**
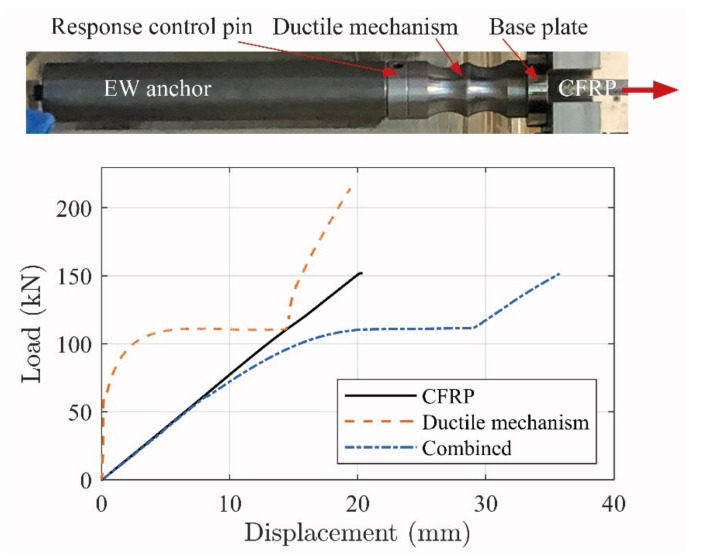
Ductile EW anchorage and related response [30].

Figure 5 shows the response of the fully assembled system (i.e., the combined tailored ductile mechanism, EW anchor, and CFRP rod) along with the individual responses of the ductile mechanism and CFRP rod. The combined response generates an initial linear elastic response and develops into a yielding regime with an approximate threshold at approximately 115 kN. This threshold was chosen to ensure that significant yielding occurred before anchor failure. When constructing the ductile mechanism, this threshold can; thus, be chosen to a desired value based on the anchorage response.

The high strength, linear elastic, and brittle behavior of the CFRP rod was converted into a similar high tensile strength response; however, with significant ductility provided by the ductile anchor [30]. The anchor response; thus, seems to provide a good basis for stress control when combined with an NSMR strengthening system. It is; however, a prerequisite that the applied ductile anchor system is activated, during the structural loading procedure, in order to ensure the desired strengthening effect. This activation does; however, not seem possible when an epoxy adhesive is used for the NSMR, since anchorage is provided by the adhesive bond line. The use of a flexible adhesive is; thus, hypothesized to provide a more even distribution of the stresses along the bond line and thus reduce the possibility of IC debonding. In addition, it is deemed to enable activation of the ductile anchor system. Consequently, two flexible adhesives were used to address the anchorage activation.

### 2.4. Material Properties

Material properties related to the characteristics of the beams and end-anchor parts are presented in Table 2. Further details about the material properties can be found in [29,30].

Flexible adhesives (PU1 and PU2) were explored in this work since they are deemed to have a significant effect on the shear-slip behavior in the bond between the CFRP and concrete adherents. Material parameters of the adhesives, PU1 and PU2, are shown in Table 3. The adhesives were chosen due to the low E-moduli and large extension at failure.

The curing days for the PU beams, when tested, ranged from 7–11 days. This was due to project time limitations and laboratory availability. Nevertheless, the manufacturer curing time data indicate that the adhesive is cured around the CFRP rod. However, with the relatively large NSMR notch in mind, this could still result in adhesives with reduced stiffness and strength in some locations (compared to the fully cured state).

PU adhesive dog bone specimens were; thus, cast in order to provide an understanding concerning the elongation of these materials, as well as the time-dependent curing influence on the material tensile behavior. This investigation was additionally done to get an indication of different behaviors in the used PU adhesives at a certain curing time. A behavior difference was important to potentially defer between these and was considered (due to the time limitations) acceptable to evaluate the ductile anchored NSMR system bonded with flexible adhesives.

**Figure 6:**
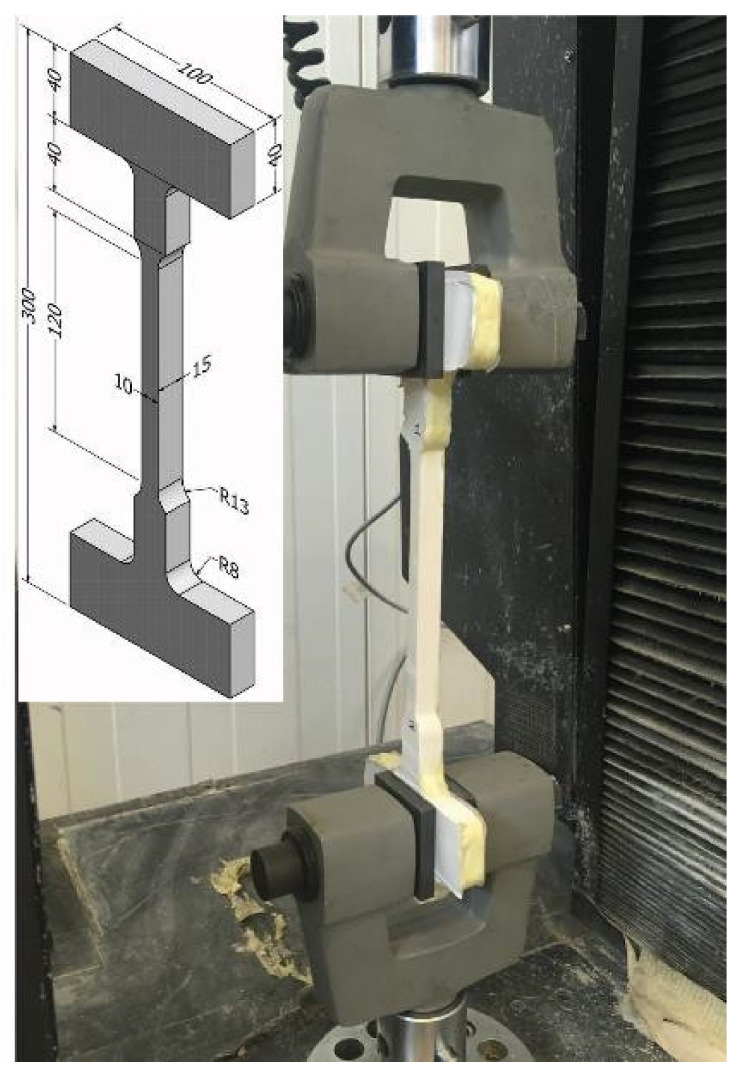
Injection of PU adhesive and related dog bone test specimen. Note: Units in mm.

The slit dimension in the concrete beams was used as a reference for the dog bone specimens, where the tested minimum cross section area was approximately 10 × 15 mm^2^. Molds made of plywood were used to cast the specimens and are depicted on Figure 6.

**Figure 7:**
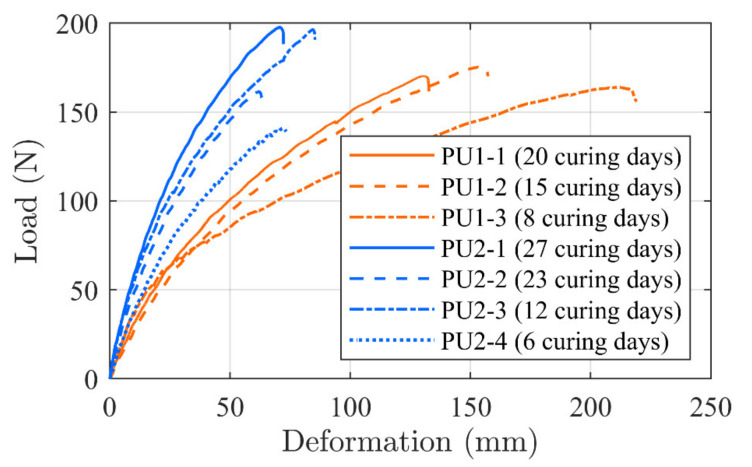
PU dog bone specimen, time related, load–deformation curves.

At least three specimens per adhesive were tested in tension at a load rate of 10 mm/min. The load–deformation curves related to each specimen are depicted in Figure 7 together with the related curing time. Note that, in test PU2-3, two curves were combined due to slippage and reloading, which do not seem to have an effect on the response.

It is seen that the curve developments provide grouped formations. This indicates that the testing method seems to provide consistency in relation to the material properties as well as the preparation method. The PU1 tests provide a maximum capacity in the interval from 140–200 N. In addition, a significant stiffness increase is seen when comparing the specimen which was cured for 20 days compared to 8 days of curing. PU1-2, which cured for 15 days, had a response curve which was located in between, but closer to the PU1-1 specimen, as expected.

The stiffness increase seems; however, to be at the expense of a reduced elongation where PU1-3 elongates more than 200 mm, while PU1-1 and PU1-2 elongate 130 and 160 mm, respectively. PU2 specimens shows more deviations in the results where two specimens provide a higher tensile value than PU1 but also an identical and lower value. However, the stiffness increases as the curing days increases. The adhesive seems to enable large elongations which, consequently, may have the desirable influence on the shear-slip bond behavior, thus enabling large deformations. It should; however, be noted that the curves provide indicational information only, since more tests at each curing day are needed to provide a verified evaluation background.

### 2.5. Test Beam Configuration and Activation

The experimental program consisted of six beams with three different strengthening configurations. In addition, an un-strengthened beam (REF) response from another experimental program [29] was included to show the actual strengthening effect. The test program and curing days related to each beam are presented in Table 4. One reference beam (PCF) was strengthened with conventional activated CFRP NSMR using epoxy resin identical to the beams tested and presented in [29]. PU1 was used as the NSMR adhesive for two beams, whereas PU2 was used for three beams. The CFRP rod in all the beams had an activation level of approximately 50% (980 MPa) of the guaranteed manufacturer CFRP strength. It shall be noted that beam PU1-1 had an activation to 44%, which was due to initial technical issues.

**Figure 8:**
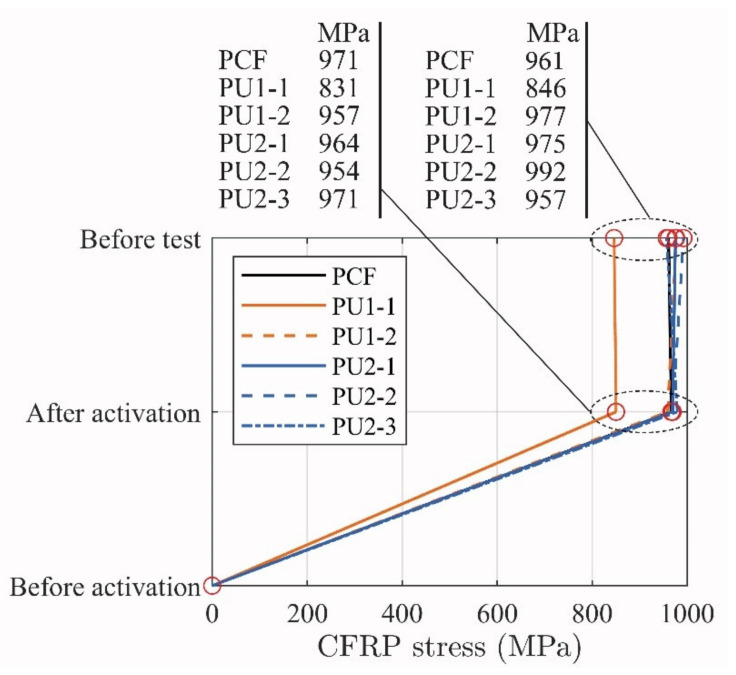
Evolution of the activation values until testing.

Figure 8 shows the stress development until testing. It should be advised that the activation took place prior to the curing of the adhesive in order to avoid initial stresses in the bonded connection.

The activation procedure was performed with the beams in an upside-down position, on the T-flange top surface. The change from activation stress did not reduce significantly during curing, which indicates a stable activation system. Thus, an additional average stress increase in the CFRP of about 21 MPa due to the beam dead load occurred in five beams when placed in its final position for testing. However, a reduction of stress occurred in the CFRP of 10 and 14 MPa PCF and PU-3, respectively. Generally, the obtained stress discrepancies from activation to before testing are small, thus not evaluated further. It should; however, be noted that future research will include further investigations of optimal adhesives used in conjunction with the ductile strengthening system.

## 3. Test Results

**Figure 9:**
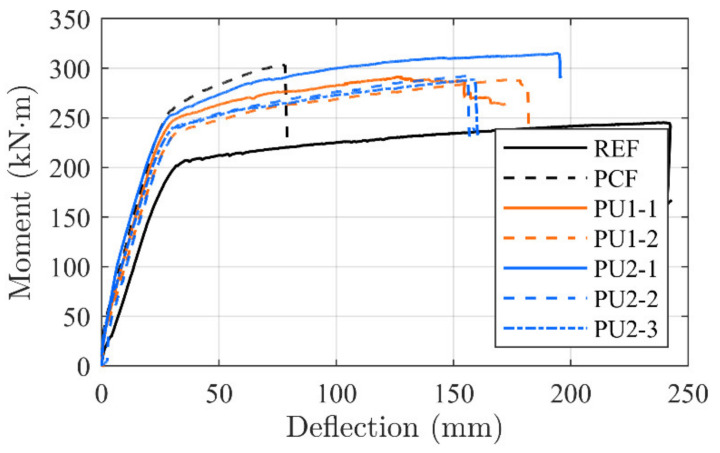
Moment–deformation test results.

Figure 9 shows the moment–deflection curves related to the tested configurations. The results are compared to a representative un-strengthened beam (REF) [29].

Crack initiation seems to occur at approximately 35 kNm for all strengthened configurations. The linear elastic cracked stage of the PU configurations continue until magnitudes of 235 to 250 kNm, and 250 kNm for PCF, where the internal reinforcement yielding point is reached. Stiffness in, what seems to be, the linear elastic cracked regime of the PU1 and PU2 configurations seems unchanged, when comparing to the conventionally epoxy-bonded NSMR strengthened reference beam (PCF).

An inclined yielding regime is achieved at this branch, which terminates at 280 to 320 kNm, where the ultimate capacity is reached. It is seen that, for all configurations, significant strengthening is gained both in the serviceability and ultimate limit state along with an increased beam stiffness. However, while for the conventional CFRP NSMR strengthened reference beam (PCF) the failure occurred at approximately 300 kNm and a deflection of 76.2 mm, in the case of PU configurations, failure took place at deflections spanning from 155 to 195 mm, corresponding to an increase up to 256% of the PCF. In addition, when isolating the yielding regime alone, an increase up to 357% was detected for the beams strengthened with the PU1 and PU2 adhesives. The brittle failure modes, which are usually seen for CFRP strengthening configurations (end or intermediate debonding), were mitigated and the governing failure mode for the PU strengthened beams was a mix of concrete crushing (CCF) in the compressive zone of the top flange of the beam and frontal overload (FO) anchorage failure (MCFO failure).

**Figure 10:**
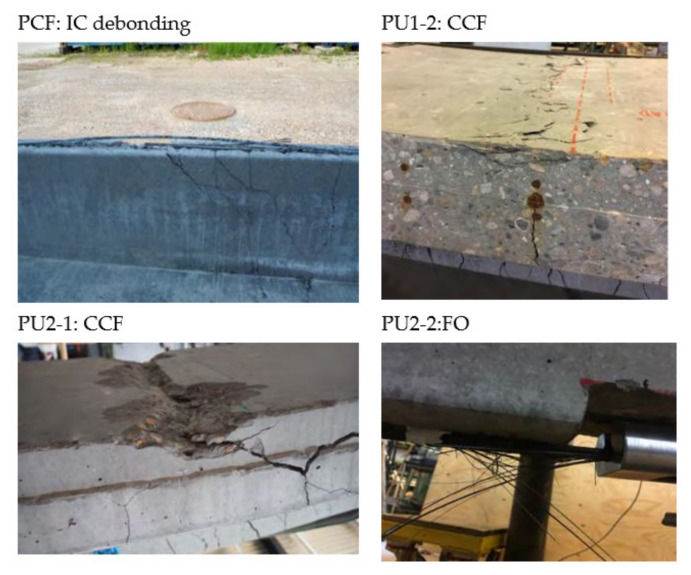
Examples of experienced failure modes.

Figure 10 shows some of the obtained failure modes. Large deflections were obtained when failure occurred. It seemed that the final stage, just before failure, reached a state which provided an equilibrium where there was an identical possibility to initiate concrete compression failure and frontal overload in the anchor. This seem to be supported by the concrete strain gauges placed at the beams center location, where all measured values at ultimate reached approximately 2.3 × 10^−3^. Consequently, the failure mode is described as MCFO (CCF or FO), to indicate this state together with the observed failure mode after testing. It should be noted that the configuration PU2-1 seems to provide a more desirable strengthening magnitude. However, one of the rolling supports was identified to have some friction during testing, which seems to result in a contributing arch effect. This effect was prevented in the other test configurations.

**Figure 11:**
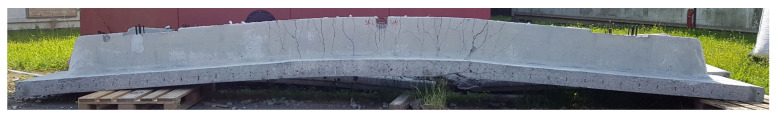
Example of deflected beam after testing.

Figure 11 shows the visual deflection of a representative overturned PU strengthened beam after testing. The permanent deformation with a large permanent deflection indicates that the yielding regime of the steel reinforcement was reached, thus providing significant warning before failure. A good correlation between the beam responses is seen. This indicates that a tailored and thus reproducible strengthening effect may be provided when applying the ductile strengthening system.

Table 5 shows the increase in deformation and yielding regime extension magnitude, when comparing the PU configurations to the PCF configuration. It is seen that a significant deformation and yielding increase are obtained when applying the ductile strengthening system.

It could be argued that the values should be compared with the un-strengthened beam. However, since a mixed concrete crushing and anchorage frontal overload failure is obtained, the concrete failure at the higher load magnitudes provide a limitation to such an evaluation. Consequently, a comparison between the well-known epoxy-bonded NSMR strengthening method used in the REF beam configurations and the PU strengthened configuration seems more suitable.

### 3.1. Activation of the Ductile Anchorage

It is hypothesized that the ductile mechanism provides significant control of the strengthening effect. A prerequisite for the desired response seems to be the interaction between the PU adhesives and the ductile EW anchors. A tailorable response is; thus, deemed to be provided by the ductile anchorages if the flexible NSMR adhesive ensures sufficient elongation and stiffness.

**Figure 12:**
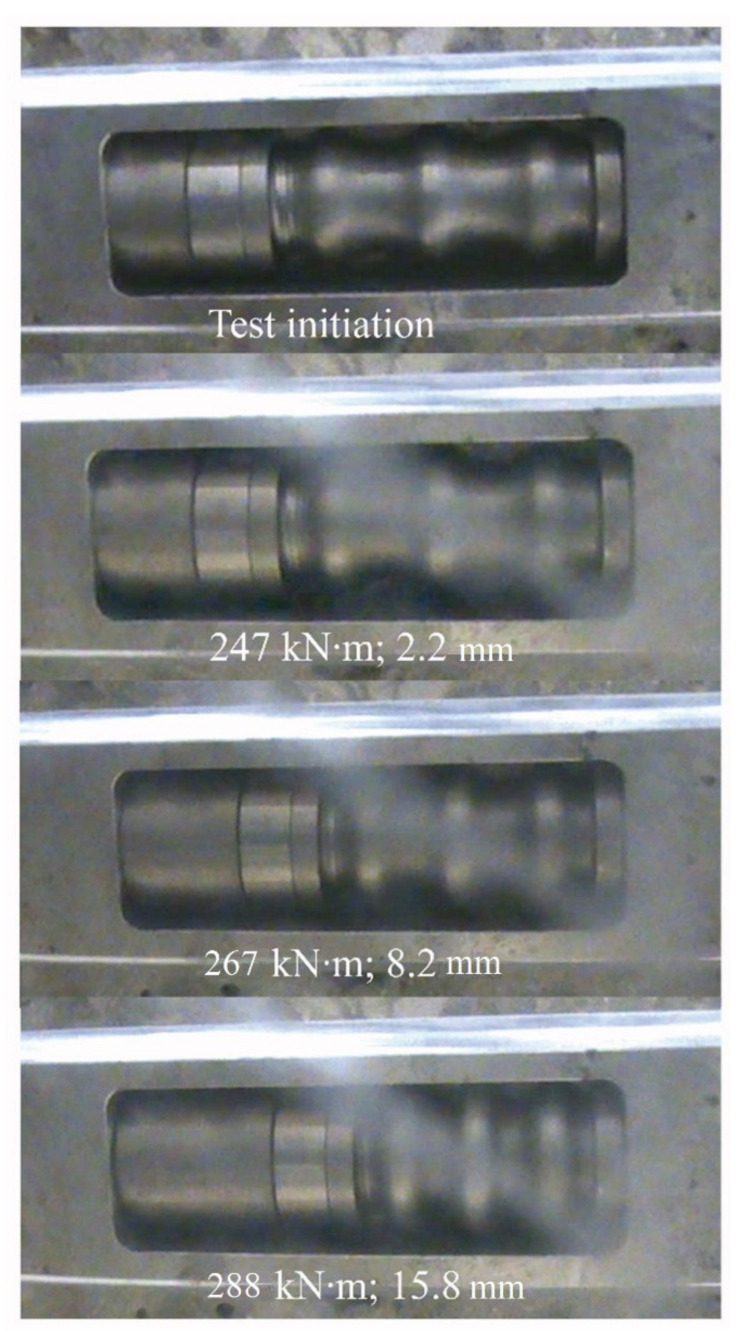
Deformation magnitudes during test of beam PU1-2.

**Figure 13:**
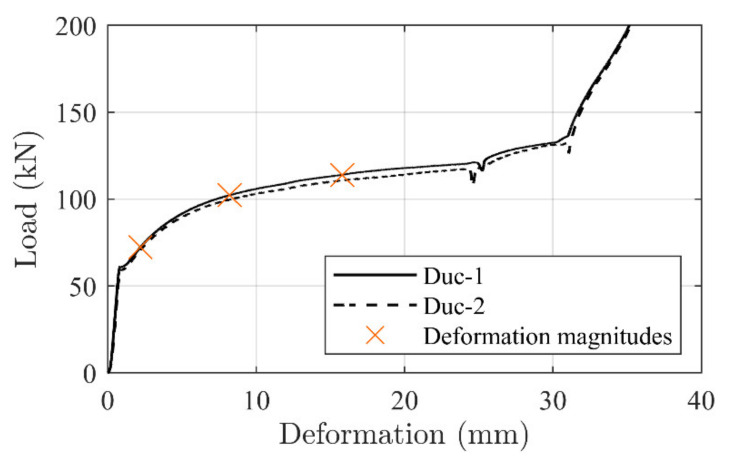
Typical complete ductile mechanism response versus ductile mechanism response in the beam PU1-2 for different stages (crosses in orange).

Figure 12 depicts a typical ductile mechanism deformation history. Four deformation magnitudes are chosen, correlating somewhat with (i) test initiation, (ii) approximate yielding point of the concrete beam, (iii) deformation at a point in the yielding regime, and (iv) ultimate capacity. It is seen that the anchorage is significantly activated, with a final deformation of 15.8 mm, when beam failure occurs. Figure 13 depicts these magnitude points and related load levels on the ductile mechanism response curve. It is seen that the ductile mechanism behaved as desired (i.e., yielding without exhausting its plastic capacity).

Table 5 shows all ductile mechanism deformation magnitudes at the left and right beam anchorage locations, as well as the summarized deformation of both ductile mechanisms from each beam configuration. It is seen from Figure 12 and Table 6 that the ductile mechanism can provide even more deformation to the strengthening system, which is not needed in the present case where MCFO failure is achieved.

### 3.2. Stress Development

**Figure 14:**
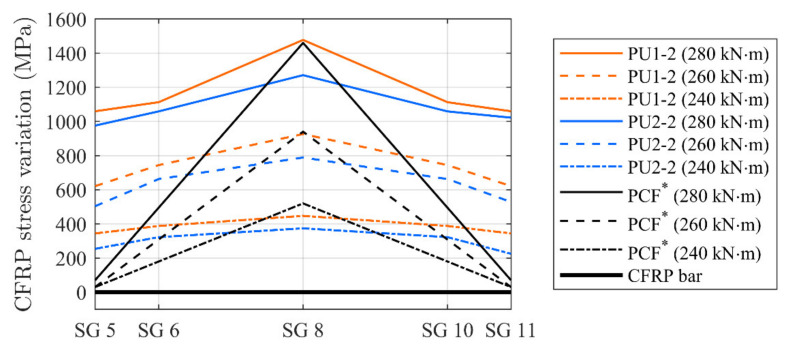
Measured stress transfer at different moment magnitudes.

Typical stress variations (without considering the activation component) at different load levels along the CFRP rod, measured by the applied strain gauges, are shown in Figure 14. A comparison to beam PCF* in [29] is included. In this case the values of the stress variations at the anchorage locations and at mid span of an activated epoxy adhesively-bonded NSMR strengthened beam are provided. Stresses at moment levels of 240 and 260 kNm and at the approximate ultimate capacity of 280 kNm are chosen to demonstrate changes in the stress development. For the case of PU beams, it is seen that the stress along the CFRP rod is almost constant until 240 kNm, but changes into a more inclined stress development at 260 kNm, increasing towards 280 kNm.

At ultimate, both flexible adhesive configurations seem to enable stress transfer in the adhesive interface between the CFRP rod and concrete. The stress transfer to the concrete beam adherent; thus, ensures an increased stress at mid span, of approximately 380 and 220 MPa for the PU1 and PU2, respectively (difference between midspan CFRP stress and stress at the anchor location).

In the case of the beam PCF*, as expected, the stress variations are marginal in the CFRP rod at the anchorage zone, since stiff epoxy does not have the same ability of stress transfer along the bond line.

**Figure 15:**
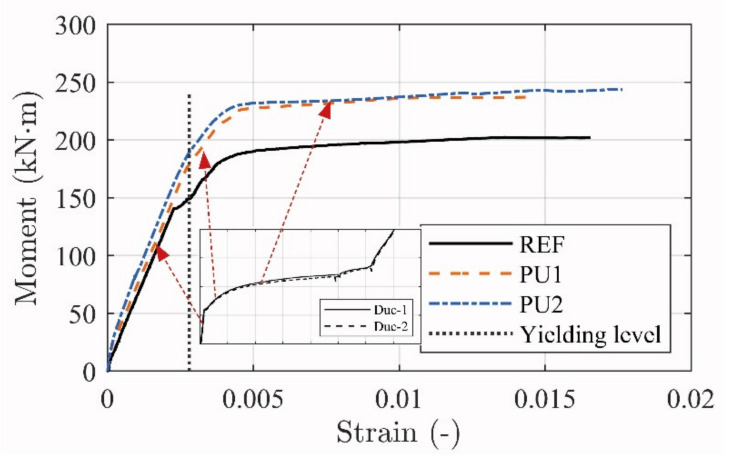
Relationship between the moment and measured strain in the steel reinforcement for PU and REF beams.

Figure 15 shows the stress development in the steel reinforcement. The ductile strengthening system seems to support the internal reinforcement well. When comparing to the un-strengthened beam REF*, the yielding thresholds are postponed and the tailored response seem to affect stresses in a controlled way until failure initiation. In addition, the strengthening support can be identified in all regimes of the beam responses, where the ductile anchorage contributes to the (i) linear elastic, (ii) yielding transition, and (iii) yielding response (see Figure 15).

The high strain magnitude resulted in malfunction of several strain gauges at approximately 0.015. Nevertheless, the data acquisition served well to show, what seems to be, a distinct response control provided by the ductile mechanisms.

The steel reinforcement stress development is slightly different when comparing the PU1 and PU2 configurations. This is deemed to be caused by the adhesive differences, where, in particular, the yielding transition regime is less pronounced for the PU2 configurations.

## 4. Analytical Predictions

The test results show that the ductile strengthening system provides the desired ductile response to the structure and the brittle IC debonding is prevented. It was shown to be possible to activate the anchorages by using the more flexible adhesive, which additionally provided a significantly lower, but more distributed, stress transfer. In addition, the stress development along the CFRP rod was monitored constant until the ultimate capacity level was reached.

**Figure 16:**
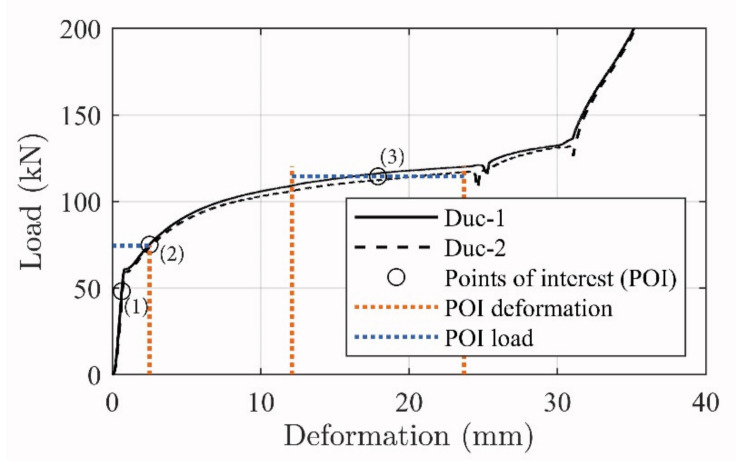
Ductile mechanism response magnitudes at the (1), (2), and (3) stages.

Figure 16 depicts the load–deformation development of the tested ductile mechanisms, where specific branches of interest are marked. These are deemed to relate to transition stages of the beam moment–deflection response curves, where (1) depicts the initial activation magnitude, (2) relates to the exposure on the ductile mechanism when yielding in the steel reinforcement initiates, and (3) is a regime from 12.1 to 23.7 mm which correspond to the minimum and maximum ductile mechanism deformation reached in the beam tests at failure (see Table 5). It is seen that the response load is horizontal and linear in the third stage, thus providing a constant load magnitude to include in the theoretical calculations.

**Figure 17:**
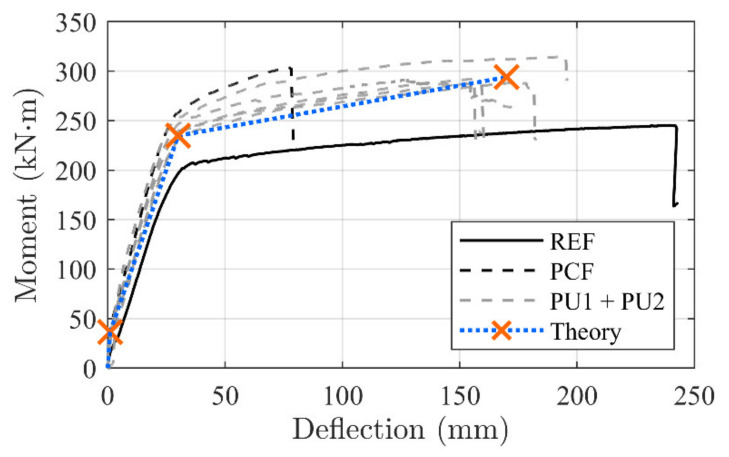
Stress variation along the CFRP bar for the different adhesives.

Due to the small stress transfer and large load distribution to the anchorage zones, it is hypothesized that the strengthening contribution can be directly related to the ductile mechanism load–deformation response. The highly controlled load transfer opens the opportunity to implement the tailored ductile mechanism load–deformation development in a conventional concrete beam evaluation approach. Figure 17 shows the moment–deflection development with the transition areas of the beam configurations (i) linear–linear cracked, (ii) yielding initiation, and, finally, (iii) the beam failure regime.

In the theoretical evaluation, the transition point between the linear uncracked and linear cracked regimes (1) can be found by evaluating the transformed cross section stresses, where the dead load moment and counteracting strengthening system moment is well known. The applied moment can then be adjusted until the bottom web stress exceeds the concrete tension stress capacity.

When evaluating the transition point of steel reinforcement yielding (2), a linear elastic cracked transformed cross section can be used for the stress evaluation. A linear relation in the steel reinforcement layers can be used to predict the CFRP strain exposure. The steel reinforcement bar closest to the beam bottom is; thus, presumed to yield.

The force exposure on the ductile mechanism can be found by performing a linear strain extension from the two steel reinforcement locations to the CFRP rod location. Following this analogy, a strain magnitude of 0.0033 was found at the CFRP location based on the estimated internal steel reinforcement strain magnitudes of 0.0029 and 0.0025 in the first and second reinforcement layer, respectively. When calculating the ductile anchorage tension force, the 7.9 mm CFRP rod cross section area and E-modulus of 165 GPa was used in the process to reach 26 kN (540 MPa). Additionally, the initial activation magnitude of 48 kN (980 MPa) was added to this value, giving a final force exposure to the ductile mechanism of 74.5 kN (see Figure 16).

It is seen that this exposure magnitude on the ductile mechanism provides a deformation of approximately 2.5 mm which correlate well with the ductile mechanism response development, depicted on Figure 11, where a deformation of 2.2 mm was measured. Consequently, this force provides a counteracting moment at the anchorage location which can be added to the original moment of the reference beam. The counter acting moment should thus be exceeded by the acting moment from the four-point bending load, before yielding occurs at an identical beam deflection magnitude as seen from the un-strengthened beam.

The ultimate failure can be found by using area (3) of the ductile mechanism (see Figure 16). It is seen that the ductile mechanism yielding regime provided at a deformation from 12.1 to 23.7 mm and thus a corresponding force of approximately 115 kN to the cross section.

The steel reinforcement tension curve (gained by tensile testing) at a deformation magnitude corresponds to the ductile mechanism, showing a stress magnitude of approximately 620 MPa, which is included in the evaluation. The internal steel reinforcement bars; thus, yield at this transition point and a cross section force equilibrium can now be utilized to estimate the ultimate capacity.

The estimated capacity is based on a constant stress in the CFRP rod between the anchorages, when using the load magnitude from the ductile mechanism response. In addition, the tests showed a CFRP rod stress increase of 380 MPa (19 kN) at the beam middle, which needs to be added to the moment capacity. Consequently, a calculated ultimate capacity of 294 kNm was found.

A deformation evaluation using the beam rotation at midspan based on the strains was used. Consequently, a summation of the concrete strain of 0.0035, deformations of both ductile mechanisms divided by the anchorage distance (equivalent ductile mechanism strain), and the CFRP strain were used.

In order to provide the rotation magnitude, the summarized strain was divided by the distance from the CFRP rod location to the top concrete surface. The deformation was achieved by applying this value to the estimate equation 1/k × rotation × support distance. k can vary from 8 to 10 depending on the load configuration applied. A k of 9 was used since the applied load contains a mixture of the load configurations. Accordingly, the deformation magnitude varies depending on the deformation changes in ductile mechanisms and adhesive transfer stress.

The theoretical concrete beam transition points (i) cracking, (ii) yielding, and (iii) failure on the moment–deflection curve, related to the PU strengthened beam configurations, are depicted in Figure 17. It is seen that a linear evolution between the transition points provide a good prediction of the response.

**Figure 18:**
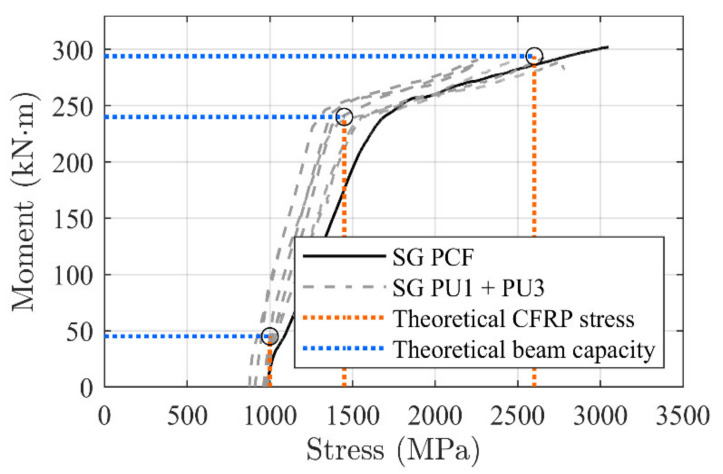
Stress variation along CFRP bar for the different adhesives.

Finally, the calculated stress magnitudes in the CFRP rod at each transition point were compared with measured values from strain gauges (5, 7 and 8). Figure 18 shows a good correlation, where a linear development between the thresholds seem to provide a good prediction. Table 7 and Table 8 provide an overview of the tested and theoretically predicted values related to the moment–deflection and CFRP stress development, respectively.

## 5. Conclusions

The presented research regards an anchored CFRP NSMR system hypothesized to provide a tailored strengthening and ductile strengthening effect. The novel ductile end anchors were used to activate 8 mm circular CFRP bars to approximately 980 MPa. Five concrete T-beams of 6.4 m (support distance 5 m) were strengthened using the flexible adhesives PU1 (two beams) and PU2 (three beams), and were compared with a conventional epoxy-bonded NSMR strengthened beam and an un-strengthened beam. It was found that the novel system provided the desired strengthening effect to the T-beams, with the following main outcomes:

- The system provided a significant and controllable strengthening effect;
- The ductile anchorages combined with the flexible adhesives ensured the desired response;
- Response curves seemed reproducible, following the tailored ductile anchor behavior well;
- Elastic regime responses of the PU configurations were similar to the one observed in the epoxy-bonded NSNR CFRP beam;
- An average deflection increase of 220% compared to the epoxy-bonded NSMR was reached;
- Average yielding regime was extended by 300% compared to the epoxy-bonded NSMR;
- Brittle delamination failure mechanisms were prevented;
- Ductile mechanism response curves could be used to theoretically predict the strengthening magnitude
- The ductile mechanism provides a measure for the applied strengthening effect, since the deformation reflects the stress in the CFRP NSMR. Consequently, it may be possible to predict the strengthening effect (CFRP NSMR stress) throughout the service life;
- A controlled yielding regime is provided by the system when the ultimate capacity is reached. The internal steel reinforcement yields as well, which means that a conventional cross section equilibrium can be performed (due to the constant force provided by the system).

The mounting procedure of the strengthening system worked well and provided a good basis for a robust activation and installation of the stressed CFRP rod. The ductile mechanisms seem to open an opportunity for extreme ductility, since several of these components can be combined and installed in extension. The novel system is currently being tested on several structural levels in order to verify the results further and facilitate a more optimized interaction between the ductile system and flexible adhesives.

## Figures and Tables

**Table 1:** Geometrical parameters of the T-beams, See Figure 2.

Parameter	Value (m)
Length of the beam, L	6.4
Distance between supports, L_s_	5.0
Free length of the carbon fiber reinforced polymer (CFRP) rod, L_f.cf_	3.9
Height of the beam, h_b_	0.47
Height of beam flange, t_f_	0.12
Width of beam web, w_w_	0.10
Width of beam flange, w_f_	0.74
Distance from top surface to outer reinforcement, d_s1_	0.42
Distance from top surface to inner reinforcement, d_s2_	0.37
Distance from top surface to CFRP rod, d_cf_	0.46

**Table 2:** Material properties related to the beam and end-anchor parts.

Parameter	Value (MPa)
Mean E-modulus of concrete **, Ec	39,000
Mean concrete cylinder compressive strength **, fcm	62
Mean yield strength of steel reinforcement **, fym	565
Mean E-modulus steel reinforcement **, Es	200,000
CFRP: Recommended ult. stress */Ult. mean stress **, fcfm	2200/3300
E-modulus CFRP tendon *, Ecf	165,000
Steel yield/tension strength of the anchor block parts *, fy/fu	235/340
Steel yield/tension strength of the threaded bar used for activation *, fy,bar/fu,bar	900/1000

Notes: * Data provided by manufacturer; ** data obtained from testing at the lab.

**Table 3:** Material properties of the flexible adhesive.

Adhesive	Parameter	Values
PU1 *	Shrinkage	3–4% [31]
	Hardness	55–60, Shore A
	Extension at failure	400% [32]
	E-modulus	1.1 MPa [32]
	Elasticity	±20%
	Temperature resistance	−40 to +90 °C
	Curing	2–3 mm/day (20 °C, AH50%)
PU2 *	Hardness	60, Shore A [33]
	Failure stress	3.0 N/mm^2^ [34]
	E-modulus	3.0 MPa
	Extension at failure	200% [34]
	Elasticity	±20%
	Curing	2 mm/day (Environment dependent)

Note: * Approximate PU material data, provided by manufacturer.

**Table 4:** Test program and activation magnitudes.

Beam	Activation (%)	Curing Days
REF ^(1)^	-	-
PCF	50	6
PU1-1	44	10
PU1-2	50	7
PU2-1	50	11
PU2-2	50	7
PU2-3	50	7

^(1)^ Beam tested in the scope of the work [29].

**Table 5:** Deformation and yielding regime extension compared to PCF configuration.

Test Configuration	Deformation	Yield Branch
	(%)	(%)
PU1-1	203	271
PU1-2	234	320
PU3-1	256	357
PU3-2	205	273
PU3-3	209	279

**Table 6:** Ductile mechanism deformation compared to ultimate moment and deflection.

Configuration	D_left_	D_right_	D_total_	Utilization	D_beam_	M_ult_
	(mm)	(mm)	(mm)	(%)	(mm)	(kN·m)
PCF	-	-	-	-	76.2	303.3
PU1-1	13.0	15.7	28.7	47.8	154.8	291.1
PU1-2	17.1	15.8	32.9	54.8	178.4	288.5
PU2-1	16.7	23.7	40.4	67.3	194.8	315.1
PU2-2	12.1	12.7	24.8	41.3	156.1	292.2
PU2-3	12.3	14.0	26.3	43.8	159.2	288.7

D_left_: Ductile mechanism deformation, at left hand side; D_right_: Ductile mechanism deformation, at right hand side; D_total_: Total deformation of both ductile mechanisms; D_beam_: Maximum beam deflection, identified in test; M_ult_: Ultimate capacity moment, identified in test.

**Table 7:** Moment and deflection predictions and test results.

Beam	Crack Initiation			Steel Yielding			Ultimate Capacity					
	M_c_	M_c,calc_	Dev	M_y_	M_y,calc_	Dev	M_u_	M_u,calc_	Dev	δ_u_	δ_u,calc_	Dev
	(kN·m)	(kNm)	(%)	(kN·m)	(kN·m)	(%)	(kN·m)	(kN·m)	(%)	(mm)	(mm)	(%)
PCF	38.7	-	-	241.2	-	-	303.3	-	-	76.2	-	-
PU1-1	37.1	35.2	5.4	233.6	234.7	0.5	291.1	293.9	0.9	155	164	5.8
PU1-2	33.5	36.5	8.9	224.3	234.7	4.6	288.5	293.9	1.8	178	172	3.5
PU2-1	35.9	36.5	1.6	237.1	234.7	1.0	315.1	293.9	7.2	195	179	8.9
PU2-2	36.9	36.5	1.1	223.1	234.7	5.2	292.2	293.9	0.6	156	153	1.9
PU2-3	37.3	36.5	2.2	231.1	234.7	1.6	288.7	293.9	1.8	159	156	1.9

M_c_: Crack initiation moment, identified in test; M_c,calc_: Calculated crack initiation moment; M_y_: Steel reinforcement yielding initiation moment, identified in test; M_y,calc_: Calculated steel yielding moment; M_u_: Ultimate capacity moment, identified in test; M_u,calc_: Calculated ultimate capacity; δ_u_: Maximum beam deflection, identified in test; δ_u,calc_: Calculated maximum beam deflection; Dev: Deviation between test and calculated values.

**Table 8:** CFRP stress predictions and test results.

Beam	Crack Initiation			Steel Yielding			Ultimate Capacity			
	*σ*_cf_	*σ*_cf,calc_	Dev	*σ*_cf_	*σ*_cf,calc_	Dev	*σ*_cf_	*σ*_cf,calc_	Dev	Failure Mode
	(MPa)	(MPa)	(%)	(MPa)	(MPa)	(%)	(MPa)	(MPa)	(%)	
PCF		1026		1666			3049			ICD *
PU1-1	875	867	0.9	1352	1476	9.2	2361	2568	8.8	UDF **
PU1-2	998	984	1.4	1369	1476	7.8	2746	2568	6.9	MCFO (CRC) ***
PU2-1	1020	984	3.6	1481	1476	0.3	2594	2568	1.0	MCFO (CRC) ***
PU2-2	912	984	7.9	1386	1476	6.4	2559	2568	0.4	MCFO (FO) ***
PU2-3	996	984	1.2	1501	1476	1.7	2442	2568	5.1	MCFO (FO) ***

* ICD: Intermediate crack debonding; ** UDF: Undefined failure (yielding threaded rods); *** MCFO: Mixed failure mode of CCF: Concrete compression failure and FO: Frontal. *σ*_cf_: Carbon fiber reinforced polymer stress (CFRP) at crack initiation, identified in test; *σ*_cf,calc_: Calculated carbon fiber reinforced polymer (CFRP) stress at crack initiation; *σ*_cf_: Carbon fiber reinforced polymer (CFRP) stress at steel reinforcement yielding initiation, identified in test; *σ*_cf,calc:_ Calculated carbon fiber reinforced polymer (CFRP) stress at steel reinforcement yielding initiation; *σ*_cf_: Carbon fiber reinforced polymer (CFRP) stress at ultimate beam capacity, identified in test; *σ*_cf,calc_: Calculated Carbon fiber reinforced polymer (CFRP) stress at ultimate beam capacity; Dev: Deviation between test and calculated values.

## Data Availability

All data and information needed for the evaluations discussed, are provided in the paper.
